# Supplementary material for: Columnar Aggregates of Azobenzene Stars: Exploring Intermolecular Interactions, Structure, and Stability in Atomistic Simulations
Source: Molecules. 2021 Dec 15;26(24):7598. doi: 10.3390/molecules26247598 (PMC8703797; doi:10.3390/molecules26247598)
Supplement: Supplementary file 1 [file molecules-26-07598-s001.zip › molecules-1511579-supplementary.pdf]

Supplementary Material for "Columnar Aggregates of Azobenzene Stars: Exploring Intermolecular Interactions, Structure, and Stability in Atomistic Simulations"

Markus Koch,[*,†] Marina Saphiannikova,[†,‡] and Olga Guskova[*,†,‡]

†*Institute Theory of Polymers, Leibniz Institute of Polymer Research Dresden, Hohe Str. 6, 01069 Dresden, Germany*

‡*Dresden Center for Computational Materials Science (DCMS), Technische Universität Dresden, 01062 Dresden, Germany*

E-mail: koch-markus@ipfdd.de; guskova@ipfdd.de

Contents

S-1 Additional Figures 3

S-2 Functional Form of the Non-Covalent Potentials 5

S-2.1 Lennard–Jones Potential (Van der Waals Interactions) 6

S-2.2 Coulomb Potential 7

S-2.3 Hydrogen Bonding Potential 7

S-3 Descriptors of the Cluster Structure 9

S-3.1 Observables, Order Parameters and Correlation Functions 9

S-3.2 Defect Detection Algorithm 10

References 11

S-1 Additional Figures

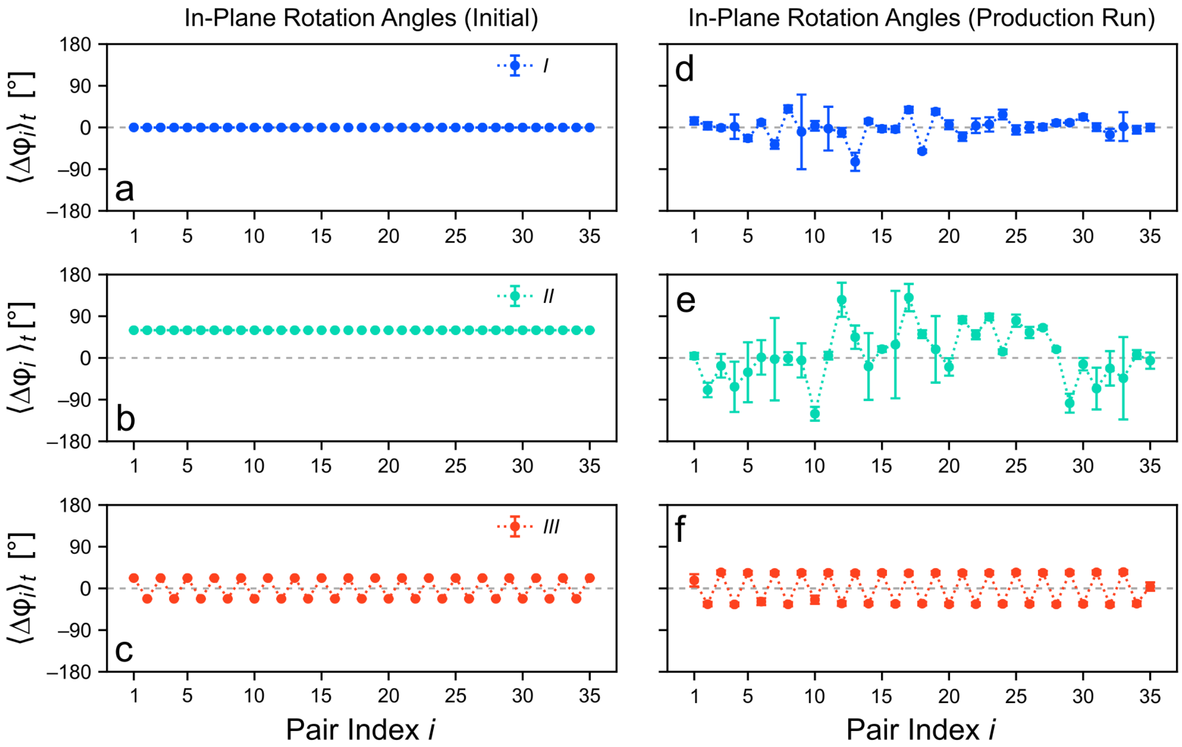

Figure S1: In-plane rotation angles of the columnar *TrisAzo* clusters (**a**–**c**) in their pre-assembled state and (**d**–**f**) after equilibration, during the production run. Time averages of $\Delta\varphi_i$ are shown as a function of the pair index i along the cluster. The rotation angles are obtained between the central phenyl rings of the BTA cores. Dotted lines serve as a guide to the eye. The oscillating in-plane rotations for type *III* stems from the pre-assembly stage. Recall that every second monomer is flipped and then slightly rotated to align the opposing azo groups.

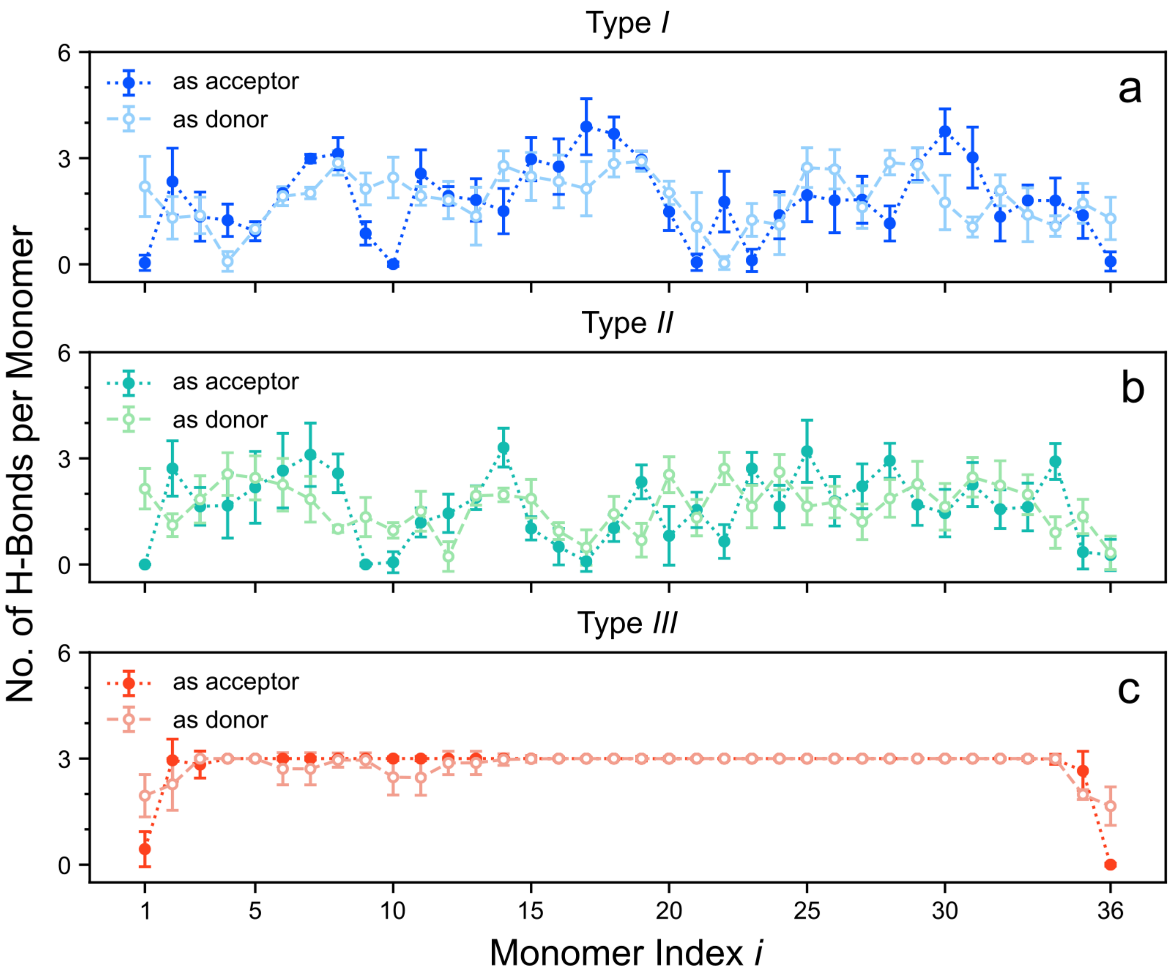

Figure S2: Number of hydrogen bonds that each monomer in the cluster participates in. Results for clusters of size $N = 36$. From top to bottom: cluster types (**a**) *I*, (**b**) *II*, and (**c**) *III*. Curves with filled (unfilled) symbols depict the number of hydrogen bonds in which the available amide groups are involved as acceptors (donors) of hydrogen. Dotted lines serve as a guide to the eye. In a few cases, unphysical hydrogen bonding scenarios are detected, e.g. *TrisAzo* molecules that participate in more than three hydrogen bonds with one neighbor.

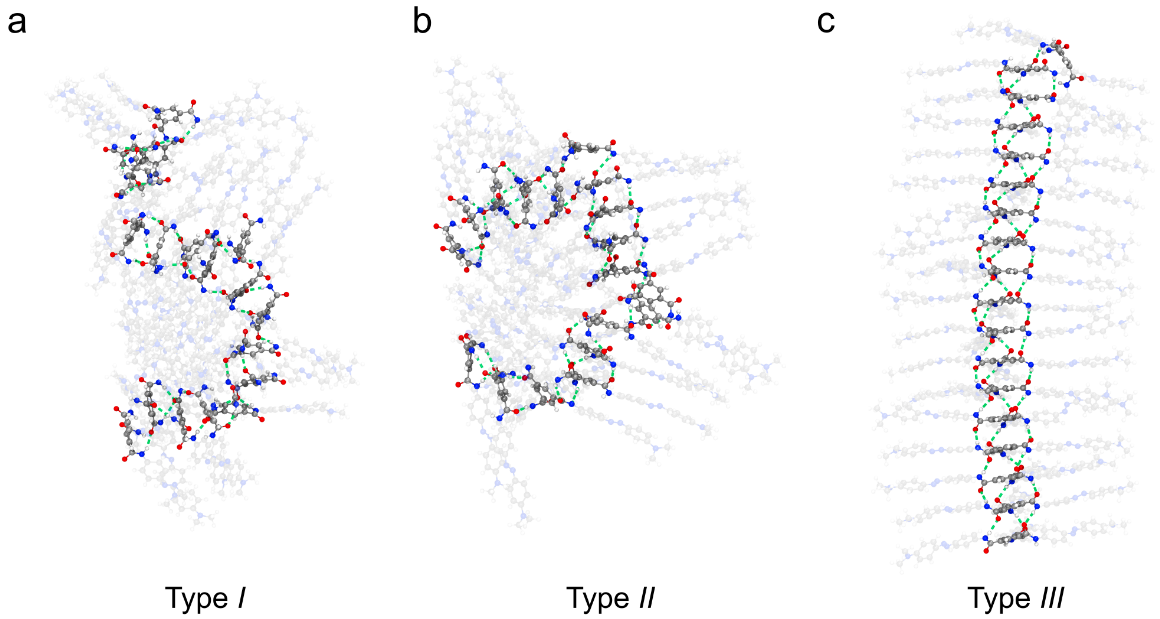

Figure S3: Simulation snapshots of the three considered cluster types with indicated hydrogen bonds (dashed green lines). From left to right: clusters of types (**a**) *I*, (**b**) *II*, and (**c**) *III*, each containing $N = 18$. The BTA groups are highlighted to enhance visibility.

S-2 Functional Form of the Non-Covalent Potentials

The following description is based on the thesis of M.K.[1] For any set of molecules, the potential energy within the DREIDING force field may be represented as a sum of two main parts, the bonded interactions E_{bonded} and the non-bonded interactions $E_{\text{non-bonded}}$.[2] The total potential energy of the system is therefore given by

$$E_{\text{pot}} = E_{\text{bonded}} + E_{\text{non-bonded}}. \tag{S.1}$$

The bonded or valence terms E_{bonded} are again a superposition of different contributions[2] (bond stretch interactions between two atoms, bond angle bend terms between three atoms, etc.).

Likewise, for the non-bonded interactions a distinction is made between electrostatic (or Coulomb) terms, E_{Coulomb}, van der Waals interactions, E_{vdW}, and explicit hydrogen bond

interactions E_{hb}.[2] The latter are not covalent bonds and are therefore included in the group of non-bonded interactions. The non-bonded part of the potential energy in DREIDING is summarized as

$$E_{\text{non-bonded}} = \sum E_{\text{Coulomb}} + \sum E_{\text{vdW}} + \sum E_{\text{hb}}. \tag{S.2}$$

Here, the sum over the first two terms is taken over atom pairs with indices $I \neq J$ (and other more specific rules, see below). For the last term, the summation is performed for triplets of eligible atoms, always involving a specifically marked hydrogen type.

S-2.1 Lennard–Jones Potential (Van der Waals Interactions)

A 12–6 Lennard–Jones (LJ) potential is employed to represent pairwise van der Waals interactions between particles, with an attractive part due to dispersion and a repulsive part resulting from electron cloud overlaps. Any pair that is spatially separated beyond a sufficiently large cutoff value r_c is not considered in the computation. The LJ potential between two eligible atoms I and J of atom types a and b separated by a distance r_{IJ} is given by

$$E_{\text{vdW}}(r_{IJ}) \equiv U_{\text{LJ}\,12\text{–}6}(r_{IJ}) = 4\epsilon_{ab}\left[\left(\frac{\sigma_{ab}}{r_{IJ}}\right)^{12} - \left(\frac{\sigma_{ab}}{r_{IJ}}\right)^{6}\right], \quad r_{IJ} < r_c. \tag{S.3}$$

The parameters ϵ_{ab} and σ_{ab} denote the depth of the potential minimum and the zero-crossing distance of the potential curve, respectively. Whereas the interaction parameters between equal atom types, e.g. aa and bb, are given in the set of force field parameters, the parameters for the non-bonded interactions between dissimilar atoms, e.g. ab and ba, need to be derived otherwise. In the present case, the Lorentz–Berthelot combination rules[3,4] are used for $\epsilon_{ab} = \epsilon_{ba}$ and $\sigma_{ab} = \sigma_{ba}$:

$$\epsilon_{ab} = \sqrt{\epsilon_{aa}\epsilon_{bb}}, \tag{S.4a}$$

$$\sigma_{ab} = \frac{\sigma_{aa} + \sigma_{bb}}{2}. \tag{S.4b}$$

S-2.2 Coulomb Potential

In atomistic force fields, the atom sites are assigned partial charges to include electrostatic effects into the model and represent different electronegativities of the atoms involved. The resulting electrostatic forces are calculated in DREIDING according to Coulomb's law.[2] The corresponding potential is given by

$$E_{\mathrm{Coulomb}}(r_{IJ}) \equiv U_{\mathrm{Coulomb}}(r_{ij}) = \frac{C}{\varepsilon_{\mathrm{r}}} \frac{q_I q_J}{r_{IJ}}, \qquad I \neq J. \tag{S.5}$$

Here, q_I and q_J are the partial charges of two atom sites I and J displaced by r_{IJ} and ε_{r} is the relative permittivity of the medium. Unless a particular implicit solvent is simulated, it is set to $\varepsilon_{\mathrm{r}} = 1$. The parameter C is a constant for energy conversion to the units of the simulation, here kcal·mol^{-1}. It contains the Coulomb constant and, thus, also the vacuum permittivity ε_0.

S-2.3 Hydrogen Bonding Potential

The DREIDING force field features an explicit hydrogen bonding potential to directly implement a hydrogen bond within a suitable group of three atoms.[2] This type of interaction involves low electronegative atoms – usually fluorine, oxygen, or nitrogen –, which play the role of electron donor (D) and electron acceptor (A). In this constellation, the donor atoms are connected to a hydrogen atom (H), for which a special atom type in DREIDING exists, marking it as eligible for hydrogen bonding. The group of three atoms then interacts via all the included non-bonded interactions (Coulomb, van der Waals) on top of the explicit hydrogen bonding potential. The latter is given by

$$E_{\mathrm{hb}}^{\mathrm{DREI}}(r_{\mathrm{AD}}, \vartheta_{\mathrm{AHD}}) = \epsilon_{\mathrm{hb}} \left[5 \left(\frac{\sigma_{\mathrm{hb}}}{r_{\mathrm{AD}}} \right)^{12} - 6 \left(\frac{\sigma_{\mathrm{hb}}}{r_{\mathrm{AD}}} \right)^{10} \right] \cos^4 \vartheta_{\mathrm{AHD}}. \tag{S.6a}$$

The distance between the donor and acceptor atoms is denoted by r_{AD}, and the angle formed by the acceptor, hydrogen, and donor atom is given by ϑ_{AHD}. The parameter ϵ_{hb} describes the depth of the potential minimum, which is located at σ_{hb} and $\vartheta_{\mathrm{AHD}} = 180°$, i.e. a linear hydrogen bond. The parameter values selected in this work are $\epsilon_{\mathrm{hb}} = 7.0\,\mathrm{kcal{\cdot}mol^{-1}}$ and $\sigma_{\mathrm{hb}} = 2.75\,\text{Å}$.[2]

The argument of the angular term is cut off at 90°, which coincides with the potential reaching zero. An inner cutoff for r_{AD} is chosen at $r_{\mathrm{in}} = 9\,\text{Å}$. To avoid jumps in the potential energy and force at the inner cutoff, the potential is extended and faded to zero within a short distance, here 2 Å, up to an outer cutoff $r_{\mathrm{out}} = 11\,\text{Å}$. For distances between r_{in} and r_{out} the potential is multiplied by a splice function $S\left(r_{\mathrm{AD}}, r_{\mathrm{in}}, r_{\mathrm{out}}\right)$, that smoothly fades the potential to zero at the outer cutoff. In the LAMMPS[5,6] implementation, this function has the form

$$S\left(r_{\mathrm{AD}}, r_{\mathrm{in}}, r_{\mathrm{out}}\right) = \frac{\left(r_{\mathrm{out}}^2 - r_{\mathrm{AD}}^2\right)^2 \left(r_{\mathrm{out}}^2 + 2r_{\mathrm{AD}}^2 - 3r_{\mathrm{in}}^2\right)}{\left(r_{\mathrm{out}}^2 - r_{\mathrm{in}}^2\right)^3}. \tag{S.6b}$$

In summary, the hydrogen bonding potential in DREIDING results as

$$E_{\mathrm{hb}}\left(r_{\mathrm{AD}}, \vartheta_{\mathrm{AHD}}\right) = \begin{cases} E_{\mathrm{hb}}^{\mathrm{DREI}}\left(r_{\mathrm{AD}}, \vartheta_{\mathrm{AHD}}\right), & r_{\mathrm{AD}} \leq r_{\mathrm{in}}, \\ E_{\mathrm{hb}}^{\mathrm{DREI}}\left(r_{\mathrm{AD}}, \vartheta_{\mathrm{AHD}}\right) S\left(r_{\mathrm{AD}}, r_{\mathrm{in}}, r_{\mathrm{out}}\right), & r_{\mathrm{in}} < r_{\mathrm{AD}} \leq r_{\mathrm{out}}, \\ 0, & r_{\mathrm{AD}} > r_{\mathrm{out}}. \end{cases} \tag{S.6c}$$

S-3 Descriptors of the Cluster Structure

S-3.1 Observables, Order Parameters and Correlation Functions

To characterize the cluster structures quantitatively, several observables and order parameters are computed. The description below is based on the thesis of M.K.[1]

Pairwise Distance

We define $\Delta r_i(t)$ as the absolute distance between the COM of two adjacent *TrisAzo* molecules (pairwise distance or stacking distance) at time t.

Local Columnar Orientation Order Parameter

The inclination angle $\Delta\alpha_i(t)$ between a pair of two neighboring *TrisAzo* molecules is defined as the angle between their orientation unit vectors $\vec{u}_i(t)$ and $\vec{u}_j(t)$, which are oriented along the shortest axes of the two oblate molecules. The vectors $\vec{u}_i$ and $\vec{u}_j$ are determined at each time step t by diagonalizing the gyration tensor of the respective molecule.[7] Then, the eigenvector corresponding to the smallest eigenvalue is selected, normalized, and identified with $\vec{u}_i(t)$ of molecule i at time t.

The so-called local columnar orientation order parameter $\Psi_i(t)$ is introduced as a local measure of the alignment between the pair i (monomers i and $i+1$). It is defined as:

$$\Psi_i(t) = |\vec{u}_i(t) \cdot \vec{u}_{i+1}(t)| = |\cos \Delta\alpha_i(t)| \,. \tag{S.7}$$

Global Columnar Orientation Order Parameter

To obtain a measure for the columnar shape of the entire cluster, $\Psi_i(t)$ is averaged over all $N-1$ neighboring pairs in a cluster of size N, resulting in $\Psi(t)$ (global columnar orientation

order parameter):

$$\Psi(t) = \frac{1}{N-1}\sum_{i=1}^{N-1} |\vec{u}_i(t) \cdot \vec{u}_{i+1}(t)| = \frac{1}{N-1}\sum_{i=1}^{N-1} |\cos \Delta\alpha_i(t)| \,. \qquad \text{(S.8)}$$

Time-averaging $\Psi(t)$ yields $\langle \Psi \rangle_t \equiv \Psi$. Note that due to the symmetry of the oblate *TrisAzo* molecules, the orientations $\vec{u}_i$ and $-\vec{u}_i$ are regarded here as equivalent. This is already implemented by taking the absolute value in Equations (S.7) and (S.8). Thus, for an ideal column $\Psi = 1$, and for random unit vectors $\Psi \approx 0.5$.

Bond–Vector Correlation Function

Another observable characterizing the cluster structure is the spatial correlation function of the orientation unit vectors, $C(|j-i|) = \langle \vec{u}_i \cdot \vec{u}_{i+|j-i|} \rangle$. Here, $|j-i|$ is the separation of two monomers i and j along the stack. The function $C(|j-i|)$ may be understood in analogy to the bond–vector correlation function known from polymer theory.[8] The orientation unit vectors $\vec{u}_i$ of the stacked *TrisAzo* monomers are used in place of the bond vectors between connected monomers in a polymer chain.

S-3.2 Defect Detection Algorithm

The locations and number of defects in columnar *TrisAzo* clusters are identified by a self-designed detection algorithm. Information about the defects is extracted from the time-averaged pairwise distances Δr_i and columnar orientation order parameters Φ_i of neighboring pairs i. Furthermore, the standard deviations σ of these variables are taken into account. A defect is identified at the position of a neighboring pair i, when at least one of the following four criteria is met:

- $\langle \Delta r_i \rangle > 6.0\,\text{Å}$,
- $\sigma(\Delta r_i) > 2.0\,\text{Å}$,

- $\langle \Phi_i \rangle < 0.5$,
- $\sigma(\Phi_i) > 0.35$.

The criteria are fine-tuned to match with the results of visual defect detection for selected cluster trajectories.

References

(1) Koch, M. The Influence of Light on a Three-Arm Azobenzene Star: A Computational Study. Ph.D. Dissertation, Technische Universität Dresden, 2022.

(2) Mayo, S. L.; Olafson, B. D.; Goddard, W. A. DREIDING: A Generic Force Field for Molecular Simulations. *J. Phys. Chem.* **1990**, *94*, 8897–8909, DOI: 10.1021/j100389a010.

(3) Lorentz, H. A. Ueber die Anwendung des Satzes vom Virial in der kinetischen Theorie der Gase. *Ann. Phys.* **1881**, *248*, 127–136, DOI: 10.1002/andp.18812480110.

(4) Berthelot, D. Sur le mélange des gaz. *C. R. Hebd. Séances Acad. Sci.* **1898**, *126*, 1703–1706.

(5) Plimpton, S. Fast Parallel Algorithms for Short-Range Molecular Dynamics. *J. Comput. Phys.* **1995**, *117*, 1–19, DOI: 10.1006/jcph.1995.1039.

(6) Thompson, A. P.; Aktulga, H. M.; Berger, R.; Bolintineanu, D. S.; Brown, M. W.; Crozier, P. S.; in 't Veld, P. J.; Kohlmeyer, A.; Moore, S. G.; Nguyen, T. D.; Shan, R.; Stevens, M.; Tranchida, J.; Trott, C.; Plimpton, S. J. LAMMPS - A flexible simulation tool for particle-based materials modeling at the atomic, meso, and continuum scales. *Comput. Phys. Commun.* **2021**, 108171, DOI: 10.1016/j.cpc.2021.108171.

(7) Ilnytskyi, J. M.; Toshchevikov, V.; Saphiannikova, M. Modeling of the photo-induced stress in azobenzene polymers by combining theory and computer simulations. *Soft Matter* **2019**, *15*, 9894–9908, DOI: 10.1039/C9SM01853K.

(8) Semenov, A. N. Bond–Vector Correlation Functions in Dense Polymer Systems. *Macromolecules* **2010**, *43*, 9139–9154, DOI: 10.1021/ma101465z.
